# Neurocognitive health in LGBTQIA+ older adults: current state of research and recommendations

**DOI:** 10.3389/fnhum.2024.1394374

**Published:** 2024-06-03

**Authors:** Riccardo Manca, Jhon Alexander Moreno, Alessandra Nicoletti, Neil J. Henderson, Jason D. Flatt

**Affiliations:** ^1^Department of Life Sciences, Brunel University London, Uxbridge, United Kingdom; ^2^Department of Medicine and Surgery, University of Parma, Parma, Italy; ^3^Department of Psychology, Université of Montréal, Montréal, QC, Canada; ^4^Centre Intégré Universitaire de Santé et de Services Sociaux du Centre-Sud-de-I'Île-de-Montréal (CCSMTL), Montréal, QC, Canada; ^5^Notre-Dame Hospital, Centre intégré universitaire de santé et de services sociaux du Centre-Sud-de-I'Île-de-Montréal (CCSMTL), Montréal, QC, Canada; ^6^Department of Medical and Surgical Sciences and Advanced Technologies, University of Catania, Catania, Italy; ^7^Department of Social Work, University of Western Cape, Bellville, South Africa; ^8^Department of Social and Behavioral Health, School of Public Health, University of Nevada Las Vegas, Las Vegas, NV, United States

**Keywords:** dementia, sexual and gender diversity, LGBTQIA+, cognitive decline, clinical neuroscience, major neurocognitive disorder

## 1 Introduction

Over the last decade, research on the health of sexual and gender minorities (SGM), i.e., lesbian, gay, bisexual, transgender, queer, and people with other sexual orientations and forms of gender expression, has been expanding to explore cognitive decline and dementia risk. This field still represents a small niche, especially when compared to research on sexual health and HIV. Indeed, data on neurological disorders in SGM populations are very scarce. For instance, a recent review of 348 neurological studies found 60 focused on cognition of SGM people, only six of which (10%) investigated cognitive health unrelated to HIV (Rosendale et al., 2021). A possible reason is due to dementia being an issue concerning primarily older people (van der Flier and Scheltens, 2005). The SGM older adult population is extremely small, although somehow difficult to quantify since most aging cohort studies collect no information about sexual orientation and gender identity (SOGI). The 2021 population census in England and Wales (Office for National Statistics, 2023) has shown that people aged 65+ are far less likely to self-identify as SGM than people aged 16–24 (<0.6% vs. 1–4%). Similar estimations were reported in other Western and primarily English-speaking countries (Wilson et al., 2020; Canada Statistics, 2022; Flores and Conron, 2023). Although difficult to assess, recent public debates, especially regarding gender minorities, might have also limited research on some SGM groups (van Anders et al., 2023).

Compared with heterosexual and cisgender groups, SGM people show higher rates of subjective cognitive decline (SCD), i.e., self-reported perception of cognitive deterioration (Romanelli et al., 2023). Although SCD is considered to be a risk factor for dementia, only a proportion of people with such diagnosis may be experiencing preclinical symptoms of a neurodegenerative disease (Jessen et al., 2020). Several factors unrelated to neurodegeneration may affect the subjective perception of cognitive decline (Ribaldi et al., 2022). This may explain inconsistencies regarding differences in objective measures of cognitive health (e.g., performance on cognitive tests and dementia risk) between SGM and non-SGM groups (Perales-Puchalt et al., 2019; Manca and Venneri, 2020; Hsieh et al., 2021; Liu et al., 2021; Saunders et al., 2021; Manca et al., 2022; Correro et al., 2023; Hanes and Clouston, 2023; Yang et al., 2024). Nonetheless, gender-diverse older adults appear to be an SGM subgroup with worse dementia risk profiles than heterosexual cisgender people (Guo et al., 2022; Brady et al., 2023; Hughto et al., 2023; Jasuja et al., 2023; Saunders et al., 2023).

As a result, it is currently not possible to draw definite conclusions on whether SGM people may have an increased risk of cognitive decline (Table 1). This knowledge gap persists even though several other health disparities (e.g., higher risk of stress, anxiety, depression, suicide, functional limitations, hypercholesterolemia, heart attack) have been extensively documented in SGM groups. These disparities have been linked to the impact of adverse social environments (Diamond and Alley, 2022). Older SGM individuals are likely to have lived through negative social conditions in their youth, since homosexuality was only removed from the Diagnostic and Statistical Manual of Mental Disorders in 1973 (Flatt and Cicero, 2023) and gender-diverse people still require a diagnosis of gender dysphoria to receive affirming healthcare. Therefore, there remains a need to investigate the possible causes or risk factors that may impact dementia risk in SGM older adult populations. This opinion paper will comment on the current state of this field to highlight current knowledge limitations and propose suggestions to promote advancements in the study of neurocognitive health of SGM populations from an interdisciplinary perspective combining clinical neuroscience, experimental and social science approaches.

**Table 1:** Current knowledge of neurocognitive health in sexual and gender minorities.

	**Sexual minorities**	**Gender minorities**
Group definitions	•Self-identified gay, lesbian, and bisexual people •Non-heterosexual groups identified based on either sexual preference or behavior •People in same-sex relationships	•Self-identified transgender and non-binary people •Two-step identification based on sex assigned at birth and current gender •People with diagnosis of gender identity disorder
Subjective cognitive health	•Inconsistent evidence of increased SCD rates (concentration may represent more of an issue than memory) •Depression, anxiety, stress, personality traits, perceived social status, ethno-racial minority status associated with higher SCD rates	•Consistent evidence of increased SCD rates •Ethno-racial minority status and depression associated with higher SCD rates
Objective cognitive health	•Possible increased dementia risk in people aged <55 •No evidence of increased risk of Alzheimer's disease dementia •Consistent evidence of better episodic memory in people aged 50+ •Poor mental and physical health, negative health behaviors, minority stress, lack of social connections, not being married, low socio-economic status, and childhood sexual abuse associated with worse cognitive health	•Consistent evidence of increased dementia risk •Higher rates of cardiovascular, physical, and mental health risk factors for dementia, but no evidence of association with cognitive health
Brain health	•No evidence of worse brain atrophy •Inconsistent evidence of negative associations between neuropsychiatric symptoms and brain structure	•No data available

## 2 Neuroscientific research

A first area of weakness is the striking lack of neuroscientific research addressing cognitive health of SGM older adults (Rosendale et al., 2021). To date, evidence on increased dementia risk is scarce and etiology is usually undetermined (Saunders et al., 2021, 2023; Guo et al., 2022; Hughto et al., 2023), thus leaving unanswered questions on whether non-neurodegenerative conditions may contribute to cognitive decline. Investigations specific to Alzheimer's disease found nine times higher risk (adjusted odds ratio = 8.95, 95% CI = 4.25–18.83) in transgender and gender diverse people (Jasuja et al., 2023), but not in older adults in same-sex relationships (Perales-Puchalt et al., 2019).

SCD is the outcome measure most commonly studied for aging SGM groups, often operationalized as a binary variable based on self-rated worsening in memory and/or thinking abilities (Seelman, 2019; Brown and Patterson, 2020; Nelson and Andel, 2020; Brown et al., 2023). Although this approach may provide useful exploratory findings to guide future investigations, the SCD diagnostic label is a poor indicator of risk of cognitive impairment if not substantiated either by biomarkers or by follow-up assessments to confirm objective cognitive decline. Analogously, quantitative investigations of objective cognitive impairment have often focused on one cognitive test score only, e.g., the Montreal Cognitive Assessment and the Telephone Interview for Cognitive Status total scores (Hsieh et al., 2021; Liu et al., 2021), and the Mental Alternation Test (an executive functioning measure) (Stinchcombe et al., 2023). As a result, vulnerability to decline in specific cognitive domains (e.g., semantic memory, visuo-spatial attention, and social cognition) has never been tested in SGM older adults.

Only two studies compared gray matter alterations in older adults with dementia in same-sex and different-sex relationships (Manca and Venneri, 2020; Manca et al., 2022). This could be due primarily to the lack of SOGI data on older adult samples. Edmiston and Juster (2022) recently highlighted a bias in neuroimaging literature that has been primarily concerned with clarifying the neural correlates of SOGI characteristics, rather than addressing clinical issues relevant to SGM people. Similarly, no studies have investigated the potential associations between brain and cognitive health of SGM older adults with any of the recently established biomarkers of neurodegeneration, e.g., neurofilament light chain, and of Alzheimer's disease pathology, e.g., amyloid beta and phosphorylated tau (Jack et al., 2018).

## 3 Study design

All studies on cognitive decline in aging SGM populations currently available have drawn their samples from cohort studies and public datasets (e.g., medical records). This strategy has enabled investigations of large samples with good statistical power. But SOGI variables are included in only a few large population-based studies (e.g., Health and Retirement Study, Behavioral Risk Factor Surveillance System, Canadian Longitudinal Study on Aging, National Health Interview Survey) that have been repeatedly exploited (Flatt et al., 2018; Seelman, 2019; Brown and Patterson, 2020; Nelson and Andel, 2020; Hsieh et al., 2021; Liu et al., 2021; Stinchcombe and Hammond, 2021, 2023; Fredriksen-Goldsen et al., 2022; Brown et al., 2023; Hopper et al., 2023; Stinchcombe et al., 2023). Possible overlaps between samples across these studies with consequences on biased estimations of SOGI-related effects on cognitive outcome measures cannot be ruled out.

Moreover, these databases might be affected by different types of bias, such as SOGI-related non-response (Jesdale, 2021), healthy volunteer (Lindsted et al., 1996), and survival biases (Cochran et al., 2016), that may affect findings and their interpretations. These may have skewed sample compositions (e.g., mostly White, highly educated, healthier participants with greater social resources) explaining both inconsistent findings on dementia risk (i.e., either higher than or equivalent to non-SGM people) and better episodic memory performance of SGM people (Stinchcombe and Hammond, 2021; Manca et al., 2022; Manca and Venneri, 2023).

## 4 SGM group definition

A high degree of methodological heterogeneity characterizes the definition of SGM groups. While most studies used self-identified sexual orientation (Seelman, 2019; Brown and Patterson, 2020; Nelson and Andel, 2020; Flatt et al., 2021; Hsieh et al., 2021; Saunders et al., 2021; Stinchcombe and Hammond, 2021, 2023; Fredriksen-Goldsen et al., 2022; Brown et al., 2023; Hopper et al., 2023; Stinchcombe et al., 2023; Yang et al., 2024), some focused on either sexual attraction (Manca and Venneri, 2023) or same-sex relationships (Perales-Puchalt et al., 2019; Manca and Venneri, 2020; Liu et al., 2021; Manca et al., 2022; Correro et al., 2023; Hanes and Clouston, 2023) due to lack of detailed SOGI data. Gender minorities have been identified by using either self-reported identity (Brady et al., 2023; Cicero et al., 2023; Saunders et al., 2023) or by selecting people with gender identity disorders who accessed gender-affirming care (Guo et al., 2022; Hughto et al., 2023; Jasuja et al., 2023). Such differences limit the generalizability and comparability of findings. Since SOGI characteristics are multidimensional, it is possible that different measurement approaches may not equally detect health disparities in SGM populations. Indeed, some accounts are available on distinct associations between sexual orientation dimensions and both mental (Bostwick et al., 2010) and physical health (Dyar et al., 2019), but not with cognitive outcome measures.

## 5 Risk profiles

While most studies have attempted to quantify cognitive health differences between SGM and non-SGM people, little research has focused on hypothesis-driven factors driving potential disparities. Correro and Nielson (2020) suggested that minority stress (Meyer, 2003) could be a crucial risk factor for cognitive decline, yet only one recent study investigated and found a negative impact of minority stress on fluid intelligence (but not on episodic memory and temporal orientation abilities) of non-heterosexual older adults (Manca and Venneri, 2023). This is primarily due to the lack of data on risk/protective factors relevant to SGM populations in cohort studies and databases not designed for such purpose. Mental health conditions (e.g., anxiety and depression) are more common among the SGM individuals probably due to a greater burden of stigmatization, victimization, discrimination and barriers to accessing healthcare (Moagi et al., 2021). Furthermore, more severe mental health issues in SGM people are consistently associated with higher risk of SCD (Flatt et al., 2018, 2021; Brown et al., 2023), cognitive impairment (Hsieh et al., 2021), and gray matter loss (Manca and Venneri, 2020). These findings suggest that some psycho-social risk factors are associated with worse cognitive health among SGM older adults. It is still unclear, instead, whether poorer mental health could have a differential impact on cognition of SGM and non-SGM older adults and, as a consequence, could lead to increased rates of dementia. By contrast, being married (Liu et al., 2021) and greater social support in lesbian women (Yang et al., 2024) seem to protect against cognitive decline, but replication of these findings is needed to draw conclusions on the extent of such effects.

## 6 Diversity

Another issue is represented by the lack of diversity in quantitative investigations of SGM cognitive health, since all current studies have been carried out in Western countries (i.e., USA, Canada, and the UK) on samples comprising mostly White English-speaking people. Although these conditions favor comparability between studies, this issue also raises concerns regarding generalizability of findings across different socio-cultural and ethno-racial contexts. Moreover, it could lead to underestimating cognitive decline risk profiles specific to SGM subgroups, e.g., people of color and other ethno-racial minorities (Flatt et al., 2018; Brown and Patterson, 2020) for whom childhood sexual trauma seems to be a stronger predictor of SCD decline than for White SGM older adults (Brown et al., 2023). When ethnicity data were collected, people of color have been systematically underrepresented in most of the studies published so far and often make up <15% of the sample. Such small sample sizes prevented clinically meaningful analyses (Perales-Puchalt et al., 2019; Manca and Venneri, 2023) and led researchers to choose race/ethnicity as a covariate to control for, rather than investigating interaction/mediation effects with SOGI characteristics (Hsieh et al., 2021; Liu et al., 2021; Correro et al., 2023).

## 7 Discussion and recommendations

Considering the limitations highlighted above, we provide a few recommendations for researchers interested in investigating neurocognitive aging and decline in SGM groups.

### 7.1 Boost neuroscientific investigations

More clinically informed studies favoring integrations of methods from clinical neuroscience and social sciences are encouraged to clarify in depth any neurocognitive health disparities between SGM and non-SGM older adults. Crucial aspects to address are the possible biological causes (i.e., dementia etiologies) driving such disparities and whether they may differ across SGM subgroups, such as transgender people who have a higher multiple sclerosis risk (Pakpoor et al., 2016). Moreover, investigating diversified indices of self-reported and objective cognitive health and biomarkers (e.g., cerebrospinal fluid and blood) will provide useful insights to clinicians to tackle neurocognitive deterioration in SGM people.

### 7.2 Improved and targeted studies

Future studies should implement appropriate and inclusive practices for collection of SOGI data, e.g., self-identification, two-step questions for gender identity, culturally appropriate choices (Flatt et al., 2022; Flatt and Cicero, 2023), that may boost opportunities to investigate health outcomes in SGM populations. Diversified study designs, such as case-control studies with smaller yet justified sample sizes could address specific questions around cognitive decline (e.g., comparing SCD vs. cognitive deficits on neuropsychological tests) and brain health in SGM populations. More targeted hypothesis-driven investigations would also improve engagement and recruitment of SGM populations in clinical trials to address overlooked issues regarding treatment response in diverse samples (Adkins-Jackson et al., 2022).

### 7.3 Impact of different SOGI dimensions

The operationalization of SOGI characteristics should be carefully considered, since comparing SGM subgroups identified using different strategies (e.g., based on self-identification, sexual behavior or medical diagnosis) may highlight unique risks for cognitive decline. Investigations with less represented SGM groups (e.g., asexual, bisexual, intersex, non-binary and ethno-racial minority people) should also be encouraged, since their health and experiences may be unique in terms of exposure to both biological and social factors. Eventually, this knowledge could provide useful evidence to train clinicians and researchers with the aim to improve research engagement of and provide much needed appropriate care to diverse SGM older adults (Moreno et al., 2017; Nicoletti et al., 2023).

### 7.4 Testing hypothesis on risk/protective factors

Although a few studies found that poor mental health may affect cognitive health in SGM groups, more investigations are needed to ascertain what factors (e.g., minority stressors) may explain heterogeneity in cognitive decline and dementia risk within SGM groups. Moreover, the impact of psycho-social factors (e.g., depression, socio-economic status, etc.) on both cognitive performance and brain metrics (e.g., volumes and functional connectivity) should be compared between SGM and non-SGM samples to clarify group-specific effects that could explain disparities. In fact, previous investigations found that depression was associated with worse cognitive performance equally in heterosexual and non-heterosexual older adults (Manca and Venneri, 2023) and that psychological distress did not explain higher rates of SCD in SGM people (Fredriksen-Goldsen et al., 2023). Future studies should also address the role of protective and resilience factors, either general (e.g., cognitive reserve (Stern et al., 2020)) or specific for SGM older adults (e.g., social support (Yang et al., 2024) and identity affirmation (Fredriksen-Goldsen et al., 2017)).

### 7.5 Improve diversity of samples

Addressing issues regarding intersectionality between SOGI and other socio-demographic determinants of health (Reygan et al., 2022) will contribute to expanding this field, potentially in a global perspective. Cross-cultural investigations of SGM older adults living across diverse countries are needed to determine what factors may influence the risk of cognitive deficits and inform collaborative public health policies. Although this may not be feasible in nations where SGM people still face challenging social environments (e.g., incarceration, violence, and stigma), potential risks for cognitive health could be ascertained in SGM populations with a history of migration and former asylum seekers.

## 8 Conclusions

In this paper we have provided an overview of the current knowledge and controversies on neurocognitive health of older SGM people and some suggestions for addressing unanswered questions. Advancing our understanding of potential challenges faced by SGM older adults and the consequences of risk/protective factors on their cognition and brain health will prove a fundamental asset to remove established barriers between health-care users and clinicians (Brooks et al., 2018) and to inform high-quality standard care practices for diverse aging populations (Rosendale et al., 2019).

## Author contributions

RM: Conceptualization, Writing – original draft, Writing – review & editing. JM: Conceptualization, Writing – review & editing. AN: Conceptualization, Writing – review & editing. NH: Writing – review & editing. JF: Conceptualization, Writing – review & editing.
