# Corrigendum: Neurocognitive health in LGBTQIA+ older adults: current state of research and recommendations

**DOI:** 10.3389/fnhum.2024.1487172

**Published:** 2024-09-10

**Authors:** Riccardo Manca, Jhon Alexander Moreno, Alessandra Nicoletti, Neil J. Henderson, Jason D. Flatt

**Affiliations:** ^1^Department of Life Sciences, Brunel University London, Uxbridge, United Kingdom; ^2^Department of Medicine and Surgery, University of Parma, Parma, Italy; ^3^Department of Psychology, Université of Montréal, Montréal, QC, Canada; ^4^Centre Intégré Universitaire de Santé et de Services Sociaux du Centre-Sud-de-I'Île-de-Montréal (CCSMTL), Montréal, QC, Canada; ^5^Notre-Dame Hospital, Centre intégré universitaire de santé et de services sociaux du Centre-Sud-de-I'Île-de-Montréal (CCSMTL), Montréal, QC, Canada; ^6^Department of Medical and Surgical Sciences and Advanced Technologies, University of Catania, Catania, Italy; ^7^Department of Social Work, University of Western Cape, Bellville, South Africa; ^8^Department of Social and Behavioral Health, School of Public Health, University of Nevada Las Vegas, Las Vegas, NV, United States

**Keywords:** dementia, sexual and gender diversity, LGBTQIA+, cognitive decline, clinical neuroscience, major neurocognitive disorder

In the published article, an author name was incorrectly written as “John Alexander Moreno”. The correct spelling is “Jhon Alexander Moreno”.

The authors apologize for this error and state that this does not change the scientific conclusions of the article in any way. The original article has been updated.

